# MRI machine learning model predicts nerve root sedimentation in lumbar stenosis: a prospective study

**DOI:** 10.3389/fneur.2025.1585973

**Published:** 2025-08-08

**Authors:** Qing Wang, Xianping Luo, Deng Li, Yi Zhai, Caiyun Ying

**Affiliations:** ^1^Chongqing Hospital of PAP, Chongqing, China; ^2^People’s Hospital of Chongqing Liangjiang New Area, Chongqing, China

**Keywords:** lumbar spinal stenosis (LSS), nerve root sedimentation sign (SedSign), magnetic resonance imaging (MRI), high-intensity zone (HIZ), epidural fat (EF), machine-learning model

## Abstract

**Objectives:**

To analyze MRI characteristics of the nerve root sedimentation sign (SedSign) in lumbar spinal canal stenosis (LSS) and to establish a risk model predicting its occurrence.

**Methods:**

A total of 1,138 narrow layers were divided into SedSign-positive (426 layers) and SedSign-negative (712 layers) groups. Key data included spinal canal diameters, dural sac dimensions, ligamentum flavum (LF) and epidural fat (EF) thickness, SedSign presence, lumbar disc herniation (LDH), high-intensity zone (HIZ), and EF classification. Comparisons used t tests or Mann–Whitney U tests. Recursive feature elimination with cross-validation (RFECV) was used to select predictive features, and models were established via random forest (RF), K-nearest neighbors (KNN), and extreme gradient boosting (XGBoost) algorithms and evaluated in terms of precision, recall, average F1 score, accuracy, and AUC. The optimal model was subject to SHAP analysis to explain the risk factors.

**Results:**

LSS patients with the SedSign had a greater degree of narrowing and were more likely to have increased EF, LDH, LF hypertrophy (LFH), and HIZ and to be older than those without the SedSign. There was no difference between the two groups in terms of sex (*p* = 0.051). RFECV yielded eight features: age, sex, APDS, APDD, TDD, EF grade, LDH, and LFH. The RF model constructed using these features—designated as SedSign8—exhibited superior performance in predicting the risk of SedSign, with robust metrics across all evaluation dimensions: precision of 84.4%, recall of 73.6%, F1 score of 78.6%, accuracy of 83.6%, and an AUC of 0.901.

**Conclusion:**

Older patients, along with a greater degree of stenosis and changes in the dural sac and surrounding tissue structures, were identified as the main pathophysiological basis for the occurrence of the SedSign in LSS.

## 1 Introduction

Lumbar spinal stenosis (LSS) is a spinal degenerative disease whose prevalence increases with age and is an important reason for spinal surgery (1–3). It is characterized by nerve compression of the lumbar spine and obvious clinical symptoms such as neurogenic claudication and pain in the lower limbs, resulting in reduced quality of life and even disability in elderly individuals. The diagnosis is usually made based on a clinical history and verified with cross-sectional imaging, like computed tomography or magnetic resonance imaging (MRI) (4). MRIis the preferred imaging examination method, as it can not only provide detailed information on the spinal canal and nerve roots and help to visualize their anatomical structure but also serve as an objective basis for surgical decision making and postoperative follow-up. At present, decompression surgery for LSS patients is often performed if conservative treatment has been ineffective or has failed, as the association between MRI-diagnosed spinal stenosis and the patient’s clinical symptoms is relatively poor (5); in other words, the decision to operate and the timing of surgery are based on the effectiveness of treatment for clinical symptoms. However, there is still no consensus on the surgical indications for LSS (6), and the technical advantages of MRI, especially in its application for LSS, still need to be further explored.

Barz et al. (7) first proposed the concept of the “nerve root sedimentation sign” (SedSign) on axial T2-weighted MRI of the lumbar spine in 2010. This sign is characterized by ‘floating’ of the majority of the cauda equina in the central or ventral part of the dural sac rather than settling in the dorsal part of the dural sac due to gravity. It was proposed that the presence of the SedSign was related to an increase in intradural pressure, which could reflect the presence and severity of nerve root compression. Subsequent research (8–11) showed that the maximum lumbar curvature, the height of the intervertebral space, the type of spinal canal stenosis, the anteroposterior diameter of the spinal canal (APDS), the area of the dural sac, the thickness of the ligamentum flavum (LF), and the presence of lumbar disc herniation (LDH) all contribute to the occurrence of the SedSign. In patients with LSS, a greater degree of mixed stenosis and spinal canal stenosis is related to a greater positive rate for the SedSign. Therefore, by assessing the distribution of the cauda equina nerves in the lumbar spinal canal, it is possible to classify and grade LSS and to differentiate between symptomatic and asymptomatic LSS (12, 13). Studies (7) have also shown that the SedSign can affect the effectiveness of conservative treatment measures for LSS. Specifically, patients with the SedSign have poorer outcomes with conservative treatment but better outcomes with surgical treatment when compared to SedSign-negative patients. This finding indicates that SedSign positivity is associated with a more severe form of stenosis and, due to the relationship with poorer treatment outcomes, a need for surgical timing. Therefore, the SedSign is highly important for guiding conservative treatment planning and the timing of surgical treatment for patients with symptomatic LSS.

In addition, recent studies involving lumbar MRI in LSS patients have increasingly focused on the high-intensity zone (HIZ), which indicates intervertebral disc degeneration and inflammatory changes, as well as spinal epidural lipomatosis (SEL). Sima S (14) proposedthat the HIZ is related to chemical radiculitis, an inflammatory condition of the nerve root caused by rupture of the annulus fibrosus, leakage of the nucleus pulposus outside the intervertebral disc, and diffusion along the nerve root. This can lead to nerve root adhesion and can serve as an imaging marker of low back pain in LSS patients. Additionally, although lumbar epidural fat (EF) is an important structure for maintaining vertebral structure and movement, SEL (15) is a well-known contributor to LSS. However, no reports have investigated whether there is a correlation between the occurrence and development of these two conditions and the SedSign.

With the advancement of big data technologies, machine learning (ML) is being integrated into the medical field with unprecedented depth and breadth (16–18). Spanning from basic medical research to clinical practice, and from disease prediction to precision medicine, its applications permeate multiple disciplines including statistics, imaging diagnostics, and molecular biology (19, 20). In medical statistics, ML has overcome the limitations of traditional analytical methods by processing massive clinical datasets through complex algorithms. It effectively resolves nonlinear relationships in intricate medical data while enabling automated feature selection, thereby deeply uncovering disease pathogenesis patterns, therapeutic efficacy, and prognostic determinants. Compared with deep learning approaches (21, 22), ML demonstrates lower requirements for data source diversity, image quality, and computational hardware performance, significantly reducing technical barriers to implementation and enhancing clinical accessibility. However, current research shows limited progress in the quantitative analysis of phenotypic data and its systematic integration with clinical workflows, with relevant research achievements and practical application cases remaining inadequately explored and documented.

Therefore, this study considers the APDS, transverse (TDD) and anteroposterior diameters of the dural sac (APDD), LDH, HIZ, LF thickening, and SEL as multiple independent variables and the presence of the SedSign as the dependent variable to develop and validate an interpretable machine learning model for identifying the risk factors for the occurrence of the SedSign in patients with LSS. The ultimate goal of this model is to improve treatment strategies through the integration of MRI findings in LSS patients by enabling early diagnosis and early treatment and to improve patient outcomes through more targeted treatment methods, thereby optimizing the management of LSS.

## 2 Materials and methods

### 2.1 Subjects

The study was approved by the Medical Ethics Committee of the People’s Hospital of Chongqing Liangjiang New Area (No: 27). The committee waived the need for informed consent because of the observational nature of this study. A total of 5,977 patients who underwent MRI examinations at our hospital between January 2022 and December 2023 were prospectively enrolled. The recruitment process commenced in January 2022 and concluded in December 2023, during which all eligible patients were identified in real - time.

The inclusion criteria were as follows (13): (1) The presence of clinical symptoms of spinal stenosis, such as neurogenic claudication or pain radiating bilaterally to the lower limbs. (2) LSS was diagnosed by clinical and MRI examinations, and the narrowed levels are at the L3/4 and L4/5 intervertebral disc levels.

The exclusion criteria were as follows: (1) patients with the most stenotic level in L5/S1; (2) Degenerative lumbar spondylolisthesis, with a slip ≥3 mm verified in lateral view. (3) Presence of a lumbosacral scoliosis of more than 20 degrees, verified on AP-view. (4) Patients with a history of trauma fracture, spinal infection, tumor and surgery.

### 2.2 MRI imaging

A SIEMENS 1.5 T MRI scanner was used, with a spinal matrix coil selected, and the subject was placed in a supine position with the lower limbs straightened and head elevated, which is the conventional position for MRI of the lumbar spine. The scanning range included the upper edge of the T12 vertebra to the level of the lower edge of the S2 vertebra. The lumbar spine MRI scanning sequences and scanning parameters were set as follows: sagittal T2WI (repetition time (TR) of 2,800 ms, echo time (TE) of 89 ms, matrix of 320 × 240), sagittal T1WI (TR of 645 ms, TE of 11 ms, matrix of 320 × 256), and sagittal fat-suppressed T2WI sequence (TR of 3,800 ms, TE of 70 ms, matrix of 256 × 192). The above sequences were performed with a layer thickness of 4 mm, layer spacing of 0.1 mm, and a field of view (FOV) of 300 mm × 300 mm; cross-sectional T2WI (TR of 2,900 ms, TE of 83 ms, matrix of 320 × 224) was performed with a layer thickness of 4 mm, layer spacing of 0.1 mm, and FOV of 300 mm × 300 mm.

### 2.3 Acquisition of relevant indicators

Relevant indicators included the APDS, TDD and APDD, LF thickness, thickness of the lumbar EF at the L3/4 and L4/5 intervertebral disc levels and the presence of the SedSign, intervertebral disc herniation, and HIZ; and the cross-sectional classification of increased EF on T2-weighted MR imaging.

#### 2.3.1 SedSign evaluation

**Figure 1:**
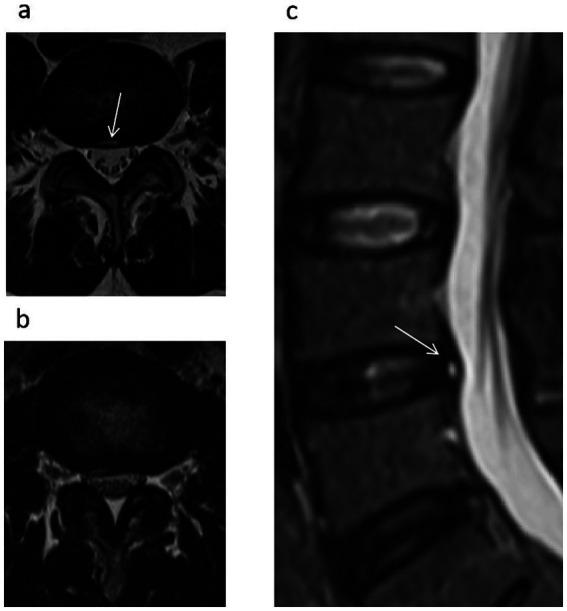
SedSign: **(a)** Negative; **(b)** Positive. T2-weighted axial **(a)** and sagittal **(c)** images demonstrating an HIZ within the central posterior annulus at the L4/5 level as indicated by the white arrow.

A negative SedSign was defined as nerve roots (other than the two exiting nerve roots) settling into the dorsal dural sac, whereas a positive SedSign was considered when the nerve roots were suspended or dispersed within the middle of or even reached the ventral side of the dural sac (7). Because the S1 and S2 nerve roots exit the dural sac ventrally, they do not show the dorsal settlement. Thus, the L5/S1 segment should be excluded when assessing these signs. Nerve root sedimentation was evaluated on axial MR images at the disc level. The presence of the SedSign was first assessed by two authors; in cases of inconsistency, a senior radiologist was consulted to reach a final assessment (Figure 1).

#### 2.3.2 TDD

**Figure 2:**
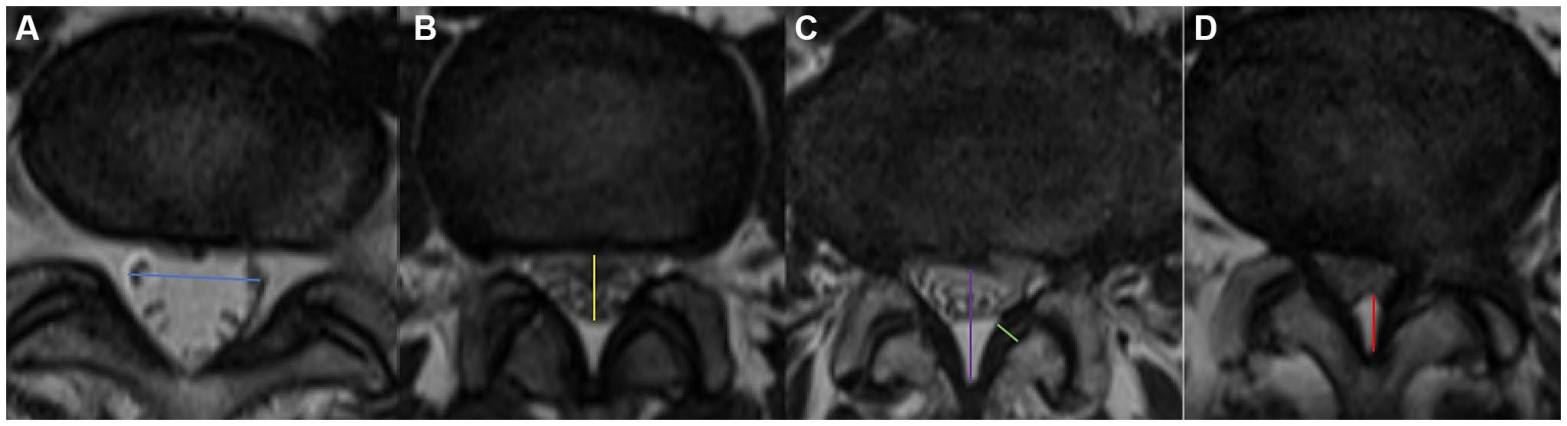
Visualization of the measurement of blue arrow the TDD **(a)**, yellow arrow the APDD **(b)**, purple arrow the APDS **(c)**, green arrow the LF thickness, and red arrow EF. Axial appearance of EF on T2-weighted MR images. The degree of fat accumulation at each intervertebral disc level was classified as follows: absent **(a)**, concave **(b)**, flat **(c)**, or convex **(d)**.

Measured at the intervertebral disc level by drawing a line between the left and right edges of the dural sac (Figure 2a).

#### 2.3.3 APDD

Measured at the intervertebral disc level by drawing a line between the anterior and posterior edges of the dural sac (Figure 2b).

#### 2.3.4 APDS

Measured at the disc level by drawing a line between the posterior border of the disc and the LF at the midline (23) (Figure 2c). We divided LSS patients into three groups on the basis of the APDS value: abnormal: APDS between 12 mm and 15 mm; narrow: APDS between 10 mm to 12 mm; and extremely narrow: APDS less than 10 mm.

#### 2.3.5 LF thickness

Measured at the midpoint of the mediolateral line of the LF perpendicular to its long axis (Figure 2c). LF hypertrophy (LFH) was defined as a thickness of 4.0 mm or greater on either side of the LF bilaterally, whereas nonthickening was defined as a thickness of less than 4.0 mm on both sides.

#### 2.3.6 HIZ

Small, circular or linear localized high-signal areas at the posterior edge of the lumbar intervertebral disc on sagittal and transverse planes on T2-weighted MR imaging (Figure 1, indicated by the white arrow).

#### 2.3.7 Lumbar EF

##### 2.3.7.1 Increased cross-sectional EF classification (qualitative)

Ishikawa et al. (24) classified the fat behind the dural sac into categories 0–3 on the basis of the amount of fat and the degree of compression on the dural sac on cross-sectional MR images (Figure 2). According to whether the dural sac was deformed by the compression, we divided these categories into Group A (no compressive deformation; categories 0 + 1) and Group B (compressive deformation, categories 2 + 3).

##### 2.3.7.2 MRI-based grading system for SEL (quantitative)

In 2003, Borré et al. (25) proposed a 3-point grading system based on the ratio of the diameter of the dural sac to the diameter of the EF as well as on the ratio of the diameter of the EF to the diameter of the spinal canal. These grades were further divided in this study into Group C (normal, or grade 0) and Group D (grade I + II + III) on the basis of the presence of fat overgrowth, as shown in Table 1.

**Table 1:** MRI grading system of SEL proposed by Borré et al. (25).

MRI Grade	APDD/EF Index	EF/APDS (%)	Meaning
Normal (Grade 0)	≥1.5	≤40	Normal amount of EF
Grade I	1.49–1	41–50	Mild overgrowth of EF
Grade II	0.99–0.34	51–74	Moderate overgrowth of EF
Grade III	≤0.33	≥75	Severe overgrowth of EF

MRI, Magnetic resonance imaging; SEL, Spinal epidural lipomatosis; APDD, Antero-posterior diameter of dural sac; EF, Epidural fat; APDS, Anteroposterior diameter of spinal canal.

### 2.4 Statistical methods

#### 2.4.1 Statistical analysis

Data were analyzed using the stats library in Python (v3.9.18). Continuous data that conformed to a normal distribution are described as M ± SD and were compared between groups with the t test; categorical data are expressed as frequencies (percentages) [n (%)]; unordered categorical variables were compared with the chi-square test or Fisher’s exact test, and ordered categorical variables were compared using the Mann–Whitney U test. *p* < 0.05 was used to indicate statistically significant differences.

#### 2.4.2 Feature selection and model construction

Feature selection and model training were performed using Python (3.9.18). The sklearn.preprocessing package was used to perform one-hot encoding on multiclass data and standardize continuous variables.

Recursive feature elimination with cross-validation (RFECV) was used for feature selection on the basis of the average accuracy after 5-fold cross-validation. With SedSign as the dependent variable, the selected features served as independent variables to create a new dataset D for model construction and analysis.

Dataset D was divided into a training set and a validation set at a 7:3 ratio for model training and validation, respectively. The sklearn.ensemble package was used to build a random forest model (RF), the sklearn.neighbors package was used to build a K-nearest neighbor (KNN) classification model, and the xgboost package was used to build an extreme gradient boosting (XGB) classification model. The sklearn.metrics package was used to validate and evaluate the constructed models; the optimal model was selected on the basis of precision, recall, F1 score, accuracy, and area under the receiver operating characteristic (ROC) curve (AUC); and the features that contributed the most to model performance were visualized with the SHAP package.

## 3 Results

Before the experiment began, two deputy chief physicians and one attending physician, all with more than 15 years of clinical experience, standardized the methods for measuring the relevant imaging parameters. The acquired measurement data were independently evaluated and measured by the two deputy chief physicians on the hospital picture archiving and communication system (PACS) and the interrater Intraclass Correlation Coefficient (ICC) was calculated to be 0.88, indicating an excellent level of agreement between the two raters.

### 3.1 Univariable analysis

A total of 1,138 patients with LSS were included following application of the inclusion and exclusion criteria, among whom 211 (18.5%) had LSS at the L3/4 intervertebral disc level, and 927 (81.5%) had LSS at the L4/5 intervertebral disc level. The patients included 508 males (44.6%) and 630 females (55.4%), with an age range of 14 to 91 years and an average age of 55.06 ± 14.19 years. The patients were subsequently divided into a SedSign-positive group [426 patients (37.4%), 206 male (48.4%) and 220 female (51.6%), average age 58.29 ± 13.952 years] and a SedSign-negative group [712 patients (62.6%), 302 male (42.4%) and 410 female (57.6%), average age 53.13 ± 13.993 years].

The relationships between sex, age, LSS level, APDS, degree of stenosis grouping, APDD, TDD, increased EF category, and the presence of LDH, HIZ, and LFH and the presence of the SedSign were assessed. The results revealed that compared with SedSign-negative patients, SedSign-positive patients were older; had smaller APDS, APDD, and TDD values; and more frequently presented with LSS at the L3/4 intervertebral disc level, LDH, an HIZ, and LFH (*p* < 0.05). Additionally, the increased EF classification of the two groups was also statistically significant (*p* < 0.001); however, the sex distribution of the two groups was marginally significantly different (*p* = 0.051). See Table 2 for details.

**Table 2:** One-way analysis of bariance of SedSign (*n* = 1,138).

Variant	Total ($\bar{X}$±s)/n (%)	Without-SedSign ($\bar{X}$±s)/n (%)	With-SedSign ($\bar{X}$±s)/n (%)	t/$\chi^{2}$/U value	*p* value
Sex				3.807	0.051
Male	508 (44.6%)	302 (42.4%)	206 (48.4%)		
Female	630 (55.4%)	410 (57.6%)	220 (51.6%)		
Age	55.06±14.194	53.13±13.993	58.29±13.952	−6.030	< 0.001
LSS level				4.207	0.040
L3/4 intervertebral disc level	211 (18.5%)	119 (16.7%)	92 (21.6%)		
L4/5 intervertebral disc level	927 (81.5%)	593 (83.3%)	334 (78.4%)		
APDS (mm)	12.85±1.593	13.28±1.206	12.13±1.878	12.563	< 0.001
Narrowness degree grouping				127.038	< 0.001
Abnormal group	828 (72.8%)	597 (83.8%)	231 (54.2%)		
Narrow group	244 (21.4%)	101 (14.2%)	143 (33.6%)		
Absolute narrow group	66 (5.8%)	14 (2.0%)	52 (12.2%)		
APDD (mm)	8.24±2.198	9.25±1.847	6.57±1.652	24.591	< 0.001
TDD (mm)	13.58±3.303	14.77±2.804	11.58±3.108	17.832	< 0.001
MRI Grading System of SEL				79999.0	< 0.001
Normal (0)	710 (62.4%)	558 (78.4%)	152 (35.7%)		
Grade I	265 (23.3%)	128 (18.0%)	137 (32.2%)		
Grade II	156 (13.7%)	24 (3.4%)	132 (31.0%)		
Grade III	7 (0.6%)	2 (0.3%)	5 (1.2%)		
Cross-sectional classification-Increased EF				65.279	< 0.001
0	59 (5.2%)	49 (6.9%)	10 (2.3%)		
1	922 (81.0%)	605 (85.0%)	317 (74.4%)		
2	110 (9.7%)	48 (6.7%)	62 (14.6%)		
3	47 (4.1%)	10 (1.4%)	37 (8.7%)		
LDH				20.887	< 0.001
No	272 (23.9%)	202 (28.4%)	70 (16.4%)		
Yes	866 (76.1%)	510 (71.6%)	356 (83.6%)		
HIZ				9.194	0.002
No	780 (68.5%)	511 (71.8%)	269 (63.1%)		
Yes	358 (31.5%)	201 (28.2%)	157 (36.9%)		
LFH				52.261	< 0.001
No	561 (49.3%)	410 (57.6%)	151 (35.4%)		
Yes	577 (50.7%)	302 (42.4%)	275 (64.6%)		

SedSign, Nerve root sedimentation sign; LSS, Lumbar spinal stenosis; APDS, Anteroposterior diameter of spinal canal; APDD, Antero-posterior diameter of dural sac; TDD, Transverse diameter of the dural sac; MRI, Magnetic resonance imaging; SEL, Spinal epidural lipomatosis; EF, Epidural Fat; LDH, Lumbar disc; HIZ, High-intensity zone; LFH, Ligamentum flavum hypertrophy.

*p* value≤ 0.05 is statistically significant.

### 3.2 Relationships between increased EF and the SedSign

To further explore whether the increased EF was related to LSS combined with the SedSign, patients without LDH, without an HIZ, and without LFH were selected for subgroup analysis. Owing to the lack of unified imaging diagnostic criteria for SEL, we conducted statistical analyses from both qualitative and quantitative perspectives. Among this group of patients, those in the SedSign-positive group had a different distribution of the increased cross-sectional EF classification (qualitative) (*p* = 0.042) and MRI lumbar SELgrade (quantitative) from those in the SedSign-negative group (*p* < 0.001), as shown in Table 3.

**Table 3:** Distribution of patients.

Variant	Total ($\bar{X}$±s)/n (%)	Without-SedSign ($\bar{X}$±s)/n (%)	With-SedSign ($\bar{X}$±s)/n (%)	$\chi^{2}$ value	*p* value
	128	109	19		
Cross-sectional classification-Increased EF				6.028	0.042
0	12 (9.4%)	12 (11.0%)	0 (0.0%)		
1	106 (82.8%)	91 (83.5%)	15 (78.9%)		
2	10 (7.8%)	6 (5.5%)	4 (21.1%)		
MRI Grading System of SEL				−4.886	<0.001
Normal (0)	103 (80.5%)	95 (87.2%)	8 (42.1%)		
Grade I	18 (14.1%)	13 (11.9%)	5 (26.3%)		
Grade II	7 (5.5%)	1 (0.9%)	6 (31.6%)		

SedSign, Nerve root sedimentation sign; EF, Epidural Fat; MRI, Magnetic resonance imaging; SEL, Spinal epidural lipomatosis.

*p* value≤0.05 is statistically significant.

The patients were also grouped according to whether the EF was increased. The results of the analysis revealed that in both the LSS with and without the SedSign groups, significantly more patients had increased EF. Moreover, the proportion of patients with increased EF in the LSS with the SedSign group was significantly greater than that in the LSS without the SedSign group (*p* < 0.05), as shown in Table 4.

**Table 4:** Distribution of patients.

Variant	Total ($\bar{X}$±s)/n (%)	Without-SedSign ($\bar{X}$±s)/n (%)	With-SedSign ($\bar{X}$±s)/n (%)	$\chi^{2}$/Z value	*p* value
Total	128	109	19		
Cross-sectional classification-Increased EF				5.431	0.041
Group A	118 (92.2%)	103 (94.5%)	15 (78.9%)		
Group B	10 (7.8%)	6 (5.5%)	4 (21.1%)		
MRI Grading System of SEL				20.894	<0.001
Group C	103 (80.5%)	95 (87.2%)	8 (42.1%)		
Group D	25 (19.5%)	14 (12.8%)	11 (57.9%)		

SedSign, Nerve root sedimentation sign; EF, Epidural Fat; MRI, Magnetic resonance imaging; SEL, Spinal epidural lipomatosis.

*p* value≤0.05 is statistically significant.

### 3.3 Feature selection and model construction

#### 3.3.1 Feature selection

After performing RFECV with 5-fold cross-validation, the average F1 score reached its maximum value (accuracy = 0.815) when 8 features were included—age, sex, APDS, APDD, TDD, increased cross-sectional EF classification, HIZ, and LFH—and used to form a new dataset D along with the dependent variable SedSign.

#### 3.3.2 Model construction

**Figure 3:**
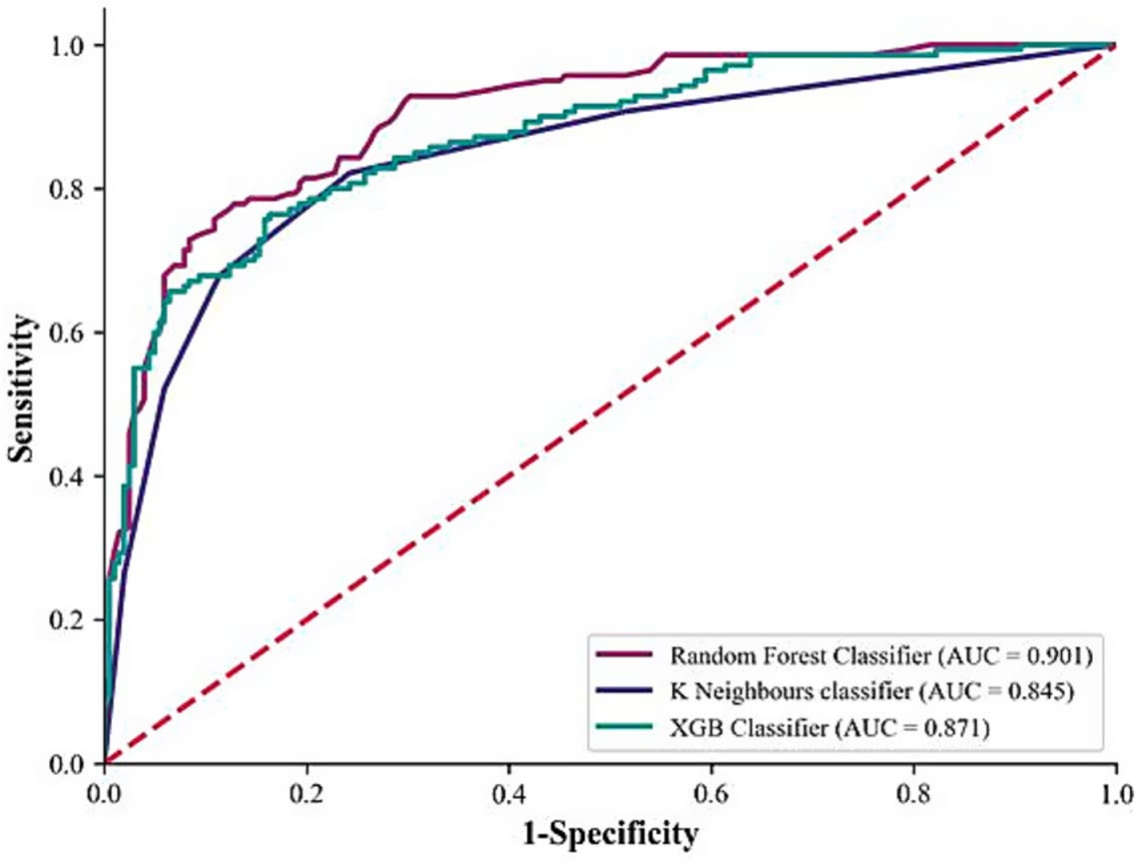
Comparison of model ROC curves.

The training set derived from dataset D was used to build the RF, KNN, and XGB classification models, and the validation set derived from dataset D was used for model validation. Among them, the RF model achieved the highest precision, recall, F1 score, accuracy, and AUC values, which were 84.4, 73.6, 78.6, 83.6%, and 0.901, respectively (Table 5; Figure 3). Consequently, the RF model was considered to have achieved the optimal performance and was named the SedSign8 model.

**Table 5:** Comparison of model evaluation metrics.

Variant	Precision	Recall	F1 Score	Accuracy	AUC
RF	0.844	0.736	0.786	0.836	0.901
KNN	0.805	0.679	0.736	0.801	0.845
XGB	0.782	0.693	0.735	0.795	0.871

AUC, Area under the curve; RF, Random Forest; KNN, K-Nearest Neighbor; XGB, eXtreme Gradient Boosting.

#### 3.3.3 SedSign8 model visualization

**Figure 4:**
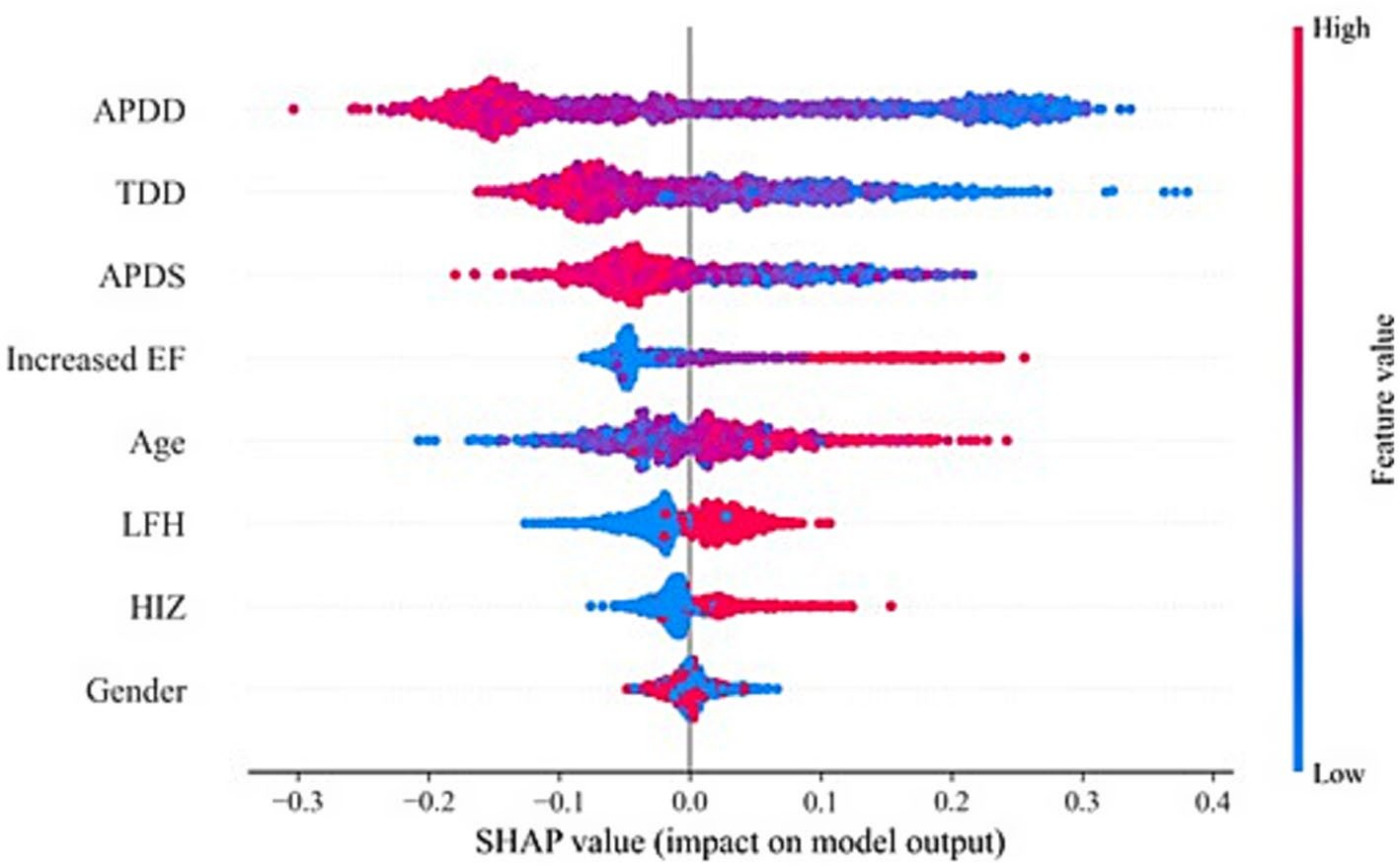
SHAP feature analysis.

**Figure 5:**
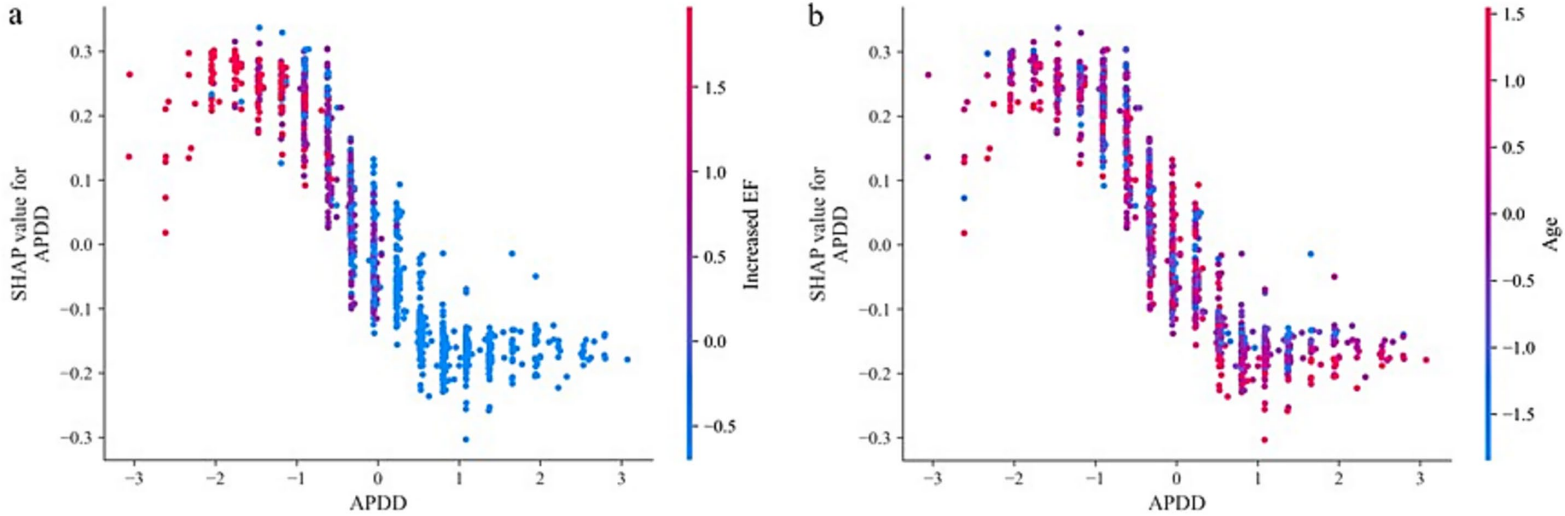
SHAP feature dependence analysis: **(a)** Dependence between APDD and EF grade **(b)**.

According to the ranking of the importance of the model features, the APDD had the greatest impact on the occurrence of the SedSign in LSS. Smaller values of the APDD, TDD, and APDS were associated with a greater likelihood of the SedSign, as were a higher EF grade and the presence of HIZ and LFH. Moreover, age was shown to be correlated with the likelihood of presenting with the SedSign. Sex also demonstrated a degree of relationship with the presence of the SedSign, as shown in Figure 4. SHAP feature dependence analysis was subsequently performed to assess the dependencies between features. Taking the APDD and EF grade as examples, smaller values of the former and larger values of the latter yielded greater SHAP values. Moreover, as the APDD decreased, the EF grade gradually increased, indicating a strong dependency between the two (Figure 5a). However, the dependence graph between the APDD measurement and age indicates a relatively weak dependency between the two (Figure 5b).

**Figure 6:**
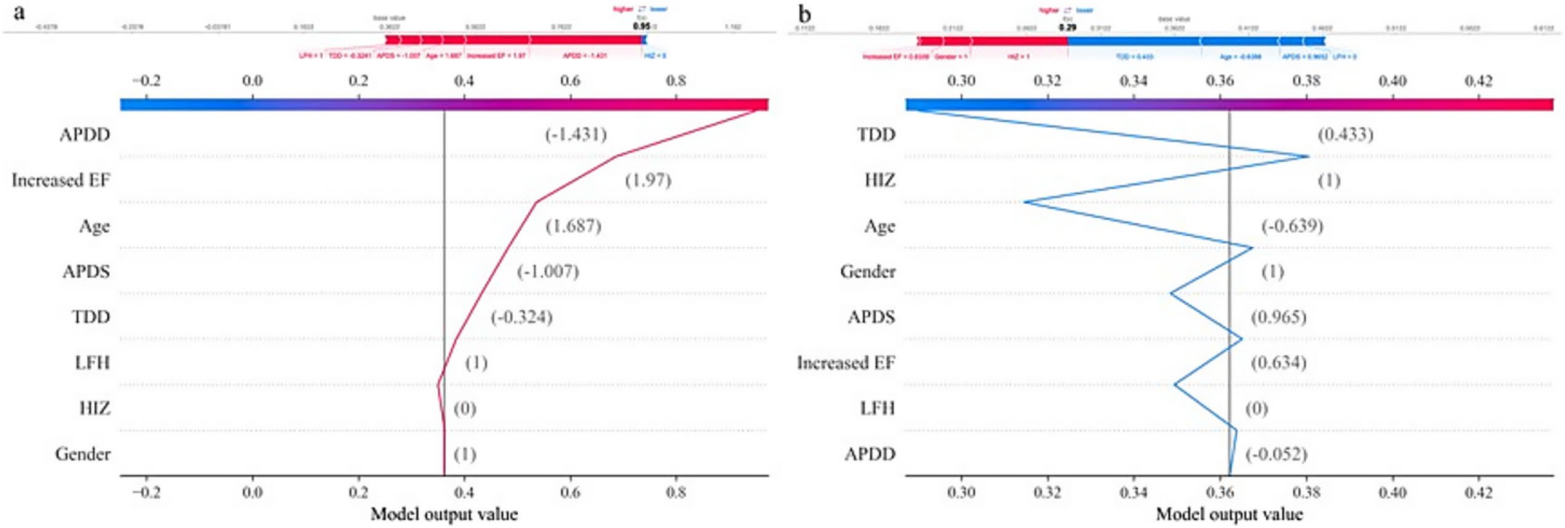
Individualized SHAP analysis: **(a)** Patient M. **(b)** Patient N.

Dependence of APDD on age. In addition, personalized risk factor alerts can be constructed on the basis of SHAP values to quantify the risk of LSS combined with SedSign. For example, Patient M is a 79-year-old male with an APDS of 11.25 mm, an APDD of 5.11 mm, a TDD of 12.51 mm, an increased cross-sectional EF classification of grade 2, without HIZ but with concurrent LFH. Although the lack of HIZ in patient M decreases his risk, the values of all other features increase his risk; overall, therefore, this patient is prone to presenting with the SedSign (Figure 6a). As another example, Patient N is a 46-year-old male with an APDS of 14.39 mm, an APDD of 8.13 mm, a TDD of 15.01 mm, an increased cross-sectional EF classification of grade 1, with concurrent HIZ but without LFH. The patient’s sex, EF grade, APDD value, and concurrent HIZ all contribute to increasing his risk, whereas his age, APDS value, TDD value, and absence of LFH contribute to reducing his risk. Overall, the patient is unlikely to experience the SedSign (Figure 6b).

## 4 Discussion

The SedSign is an MR imaging manifestation that has important clinical significance for the diagnosis of LSS, selection of treatment plans, and patient outcome prediction. The pathophysiological mechanism producing the SedSign is a complex process. First, the formation of the SedSign is closely related to the factors causing LSS; these factors lead to mechanical compression of the nerve roots, resulting in changes in local blood flow and ischemia and hypoxia of the nerve tissue as well as the initiation of local inflammatory responses, further aggravating nerve root edema and injury. Although nerves have some degree of regenerative capacity, if the compression persists, further deterioration of nerve function and even neuronal degeneration and death may occur. In summary, the pathophysiological mechanism of SedSign is a complex process involving multiple factors, such as mechanical compression, hypoxia, the inflammatory response, and changes in nerve regeneration capacity. These mechanisms provide an important theoretical basis for the clinical understanding and treatment of the SedSign.

Our research revealed that in LSS patients, older age and a greater degree of stenosis at the L4/5 intervertebral disc level increased the likelihood of developing the SedSign (*p* < 0.05). Specifically, smaller relative measurement values, such as the APDS, APDD and TDD, which reflect the degree of spinal stenosis, were related to a greater likelihood that the SedSign would occur. After grouping on the basis of the APDS, the incidence of the SedSign in the abnormal group, narrow group, and extremely narrow group was 27.9% (231/828), 58.6% (143/244), and 78.8% (52/66), respectively; in other words, as the degree of narrowing increases, the degree of mechanical compression on the dural sac and nerve roots increases, and the incidence of the SedSign significantly increases, consistent with the results of previous reports (7–10).

Our study also comprehensively assessed the MRI manifestations of the dural sac and surrounding tissue structures in patients with LSS combined with the SedSign. The results of this analysis revealed differences in LDH and LFH between the SedSign-positive and SedSign-negative groups (*p* < 0.05). Earlier studies (26–30) have shown that LDH and LFH are the main pathogenic factors affecting LSS and contribute to long-term chronic compression of the dural sac. Because LSS due to LDH and LFH is a chronic and continuous process, the SedSign is highly likely to occur in these patients. Both the anterior pressure from LDH and the lateral - posterior pressure from LFH can cause compression of the dural sac and nerve roots, and this compression contributes to the pathophysiological process underlying the formation of the SedSign.

Owing to the constraining effect of the surrounding tissues on the dural sac, the nerve roots begin to move to the ventral side and accumulate, resulting in the SedSign phenomenon. Barz et al. (7, 11–13) reported that patients with LSS and the SedSign had elevated epidural pressure during surgery, which they also considered a significant potential cause of SedSign positivity.

This study was the first to investigate the relationship between HIZ and LSS in terms of the occurrence of the SedSign. The results revealed that LSS patients with an HIZ (43.9%) were significantly more likely to present with the SedSign than those without an HIZ (34.2%). When LSS patients present with an HIZ on lumbar MRI, nearly half of them will also have the SedSign. Ren et al. (31) reported that HIZs are characterized by the formation of vascularized granulation tissue along torn annuli fibrosi; moreover, many inflammatory products are produced due to the inflammatory response, which flow out of the nerve root along the tear in the annulus fibrosus. Additionally, the inflammatory response around the dural sac may lead to fibrin exudation, forming fibrin adhesions that cause the dural sac to contract and narrow, restricting the movement of the nerve roots and preventing them from sinking at the bottom of the dural sac under the influence of gravity, resulting in the occurrence of the SedSign.

This study also revealed a significant difference in the presence of an increased EF between LSS patients with and without SedSign in LSS from both qualitative (cross-sectional classification) and quantitative (thickness grading) perspectives (*p* < 0.05). Specifically, LSS patients with the SedSign were more likely to present with an increased EF.

On MRI, the normal EF tissue behind the dural sac appears as a fat signal that runs along the spinal canal with a continuous distribution and varying degrees of deposition and primarily maintains the normal movement and function of the tissues inside the canal. The cross-sectional classification of increased EF and MRI grading of lumbar SEL can both indicate the presence of increased EF and the severity of any compression on the dural sac. On transverse MR imaging, the dural sac normally appears as a smooth, arc-shaped bulge behind the spine. As the amount of EF increases, the posterior edge of the dural sac gradually becomes flatter and more concave. Further analysis of the impact of increased EF on the occurrence of the SedSign in LSS revealed that there were significant differences among the groups (the noncompressed deformation group A, the compressed deformation group B, the nonincreased fat group C, and the increased fat group D) (*p* < 0.05). The incidence of the SedSign in the compressed deformation group (group B) and the group with increased EF (group D) reached 40%. Both EF evaluation methods indicated that increased EF is associated with the occurrence the SedSign in LSS patients (*p* < 0.01), suggesting that an increased EF is a major cause of the occurrence of the SedSign. Owing to the constraints of the bony spinal canal and ligaments, the increased EF is more likely to protrude toward the dural sac, exerting continuous pressure on its dorsal side, which leads to local blood flow changes and ischemia and hypoxia of the neural tissue, resulting in the formation of the SedSign. These findings emphasize the importance of the EF surrounding the dural sac in the occurrence of the SedSign and provide strong support for the precise and comprehensive diagnosis of LSS in the presence of the SedSign.

We innovatively integrated multi-dimensional phenotypic data to develop the SedSign8 predictive model, enabling early and precise risk stratification for SedSign-related pathologies. This model demonstrated exceptional predictive performance, achieving an AUC of 0.901, precision of 0.844, and accuracy of 83.6% on independent test datasets. Requiring only basic clinical parameters (age, sex) combined with key MRI anatomical measurements, SedSign8 generates personalized risk profiles with significant clinical utility.

In clinical workflows, following MRI examination completion, SedSign8 rapidly synthesizes patient-specific data to provide real-time risk assessment. As illustrated in Figure 6, clinicians input demographic information and MRI measurement data through an intuitive interface, prompting the system to generate dynamic line graphs that visually delineate the quantitative impact of risk modifiers and protective factors on disease progression. This facilitates immediate clinical decision-making regarding further diagnostic evaluation or therapeutic intervention.

Notwithstanding these advancements, we propose three strategic enhancements to address evolving demands in medical imaging analytics: First, we will conduct in-depth exploration of SedSign8’s latent capabilities to optimize early diagnostic pathways and therapeutic regimens. Second, we aim to develop an advanced multi-modal architecture integrating cross-sectional imaging modalities (MRI/CT/PET) and longitudinal examination data. Third, we plan to strengthen model generalizability through large-scale external validation using expanded multi-center cohorts (target n > 10,000), incorporating geographically diverse population characteristics. These initiatives will establish a new paradigm for intelligent clinical decision support systems in precision medicine.

## 5 Conclusion

In summary, for older patients, a greater degree of stenosis, and changes in the dural sac and surrounding tissue structures constitute the main pathophysiological basis for the occurrence of the SedSign in LSS patients; specifically, age, APDS, TDD, and APDD, as well as MRI parameters such as SEL, HIZ, and LFH, are key predictors of the occurrence of the SedSign in LSS patients. These findings may aid in providing a more detailed diagnostic imaging assessment for LSS, enabling better identification and management of patients with the SedSign. The SedSign8 model developed in this study can not only aid in the early and accurate diagnosis of the disease but also help in planning appropriate interventions to alleviate the disease burden and improve patient quality of life in clinical practice.

## 6 Critical relevance statement

This study employs various measurement and observation methods to reflect changes in the anatomy of the lumbar spine (particularly the cauda equina) to provide important insights for optimizing surgical decisions and improving outcomes in LSS patients.

## Data Availability

The raw data supporting the conclusions of this article will be made available by the authors, without undue reservation.
